# Addition of cyclophosphamide on insufficient response to pomalidomide and dexamethasone: results of the phase II PERSPECTIVE Multiple Myeloma trial

**DOI:** 10.1038/s41408-019-0206-8

**Published:** 2019-04-08

**Authors:** Katja C. Weisel, Christof Scheid, Manola Zago, Britta Besemer, Elias K. Mai, Mathias Haenel, Jan Duerig, Markus Munder, Hans-Walter Lindemann, Anja Seckinger, Christina Kunz, Axel Benner, Dirk Hose, Anna Jauch, Hans Salwender, Hartmut Goldschmidt

**Affiliations:** 1 Department of Oncology, Hematology and Bone Marrow Transplantation with Section of Pneumology, University Medical Center Hamburg-Eppendorf, Hamburg, Germany; 2 Department of Hematology, Oncology, Immunology, Rheumatology and Pulmonology, University Hospital of Tuebingen, Tuebingen, Germany; 3 Department I of Internal Medicine and Center of Integrated Oncology Cologne Bonn, University of Cologne, Cologne, Germany; 4 Center for Clinical Trials, University Hospital of Tuebingen, Tuebingen, Germany; 5 Department of Internal Medicine V, University Clinic Heidelberg, Heidelberg, Germany; 6 Clinic of Internal Medicine III, Hematology and Oncology, Clinic of Chemnitz, Chemnitz, Germany; 7 Department of Hematology, University Hospital Essen, Essen, Germany; 8 Department of Hematology, Oncology, and Pneumology, University Medical Center Mainz, Mainz, Germany; 9 Clinic for Haematology/Oncology, Catholic Hospital Hagen, Hagen, Germany; 10 Division of Biostatistics, German Cancer Research Center (DKFZ) Heidelberg, Heidelberg, Germany; 11 Institute of Human Genetics, University of Heidelberg, Heidelberg, Germany; 12 Department of Hematology and Oncology, Asklepios Hospital Hamburg Altona, Hamburg, Germany; 13 National Center for Tumor Diseases (NCT), University Clinic Heidelberg, Heidelberg, Germany

Treatment of multiple myeloma (MM) has continuously improved over the recent years with a number of approved novel agents resulting in prolonged progression-free (PFS) and overall survival (OS)^1^. However, patients who are refractory to proteasome inhibitors and immunomodulating agents (IMiD®) have a poor prognosis with a median OS of only 15 months^1,2^. Furthermore, with the emerging use of lenalidomide in first-line treatment, development of novel effective treatment strategies for patients refractory to lenalidomide is of critical importance. Standard treatment of pomalidomide and dexamethasone was introduced in two large phase III trials in patients with relapsed and/or refractory multiple myeloma (RRMM) with a median of 5 prior treatment lines and exposed and/or refractory to both, bortezomib and lenalidomide and refractory the last prior treatment line^3,4^. In these trials an objective response rate (ORR) of 31% and 35% and a median PFS of 4.0 and 4.2 months was reached. The addition of cyclophosphamide to immunomodulating agents demonstrated to improve efficacy regarding ORR and PFS^5,6^. Furthermore, there are clear indications that addition of cyclophosphamide may overcome IMiD® resistance^7^. Here, we report on the single-arm, phase II, multicenter, investigator-initiated German-speaking Myeloma Multicenter Group (GMMG) PERSPECTIVE trial (Eudra-CT No. 2013-003678-29) investigating the efficacy of adding cyclophosphamide to pomalidomide and dexamethasone in the case of suboptimal response after three cycles or primary progression during the first three cycles.

Sixty patients with relapsed and or refractory MM after at least two prior treatment lines including bortezomib and lenalidomide and not anymore responding to the last prior treatment were included into the trial and received pomalidomide 4 mg day 1–21 of a 28-day cycle and dexamethasone 40 mg (20 mg in patients >75 years of age) on day 1, 8, 15, and 22. The criteria for addition of cyclophosphamide in the protocol were as follows: cyclophosphamide has to be added in all patients with documented disease progression (PD) during the first three cycles (documentation of one PD event was sufficient) or all in patients not achieving at least partial remission (PR) after three treatment cycles. Cyclophosphamide was given in a dose of 500 mg/m² intravenously days 1 and 15 for a maximum of 12 cycles. Pomalidomide and dexamethasone were given until disease progression or unacceptable toxicity. Adverse events (AEs) were recorded and graded according to the National Cancer Institute Common Terminology Criteria for Adverse Events, version 4.0. Response was assessed according to the IMWG criteria^8^. Primary endpoint was to determine the ORR. Survival time (OS, PFS, second PFS (defined at PFS from start cyclophosphamide)) and time to next treatment (TTNT) distributions were estimated by the method of Kaplan and Meier^9^. Primary efficacy analysis was performed after a median follow-up time of 20.1 months; secondary objectives were analyzed after a median follow-up of 32.8 months. The intention to treat (ITT) population consisted of 59 patients. Median age was 67 years (47–81 years), median number of prior lines was 3. In total, 43.6% of analyzed patients had cytogenetic high-risk disease (del17p13, t(4;14) or >3 copies of 1q21).

ORR (≥PR) in the ITT population was 39%, which did not differ significantly from a rate of 30%, which was considered insufficient. The lower bound of the one-sided 95% confidence was 29.2%. Of the overall treated population, 14 (23.7%) patients showed a PR, 7 (11.9%) patients a very good partial remission (VGPR), and 2 (3.4%) patients a complete remission (CR). The clinical benefit rate (≥minimal remission (MR)) was 66.1% with 16 patients (27.1%) achieving a MR. In two patients, an early death occurred in or after the first cycle, both were documented as PD (Table 1).

**Table 1:** International Myeloma Working Group (IMWG) best response (ITT population)

Response	Overall (*n* = *λ* 59)	Best response cycle 1–3 or before addition of CY (*n* = 59)	Best response under POM + CY + DEX (*n* = 36)	Best response under POM + CY + DEX according to response at start CY		
				Minimal response at start CY (*n* = 5)	Stable disease at start CY (*n* = 15)	Progressive disease at start CY (*n* = 16)
Objective response rate (≥PR), *n* (%)	23 (39.0%)	11 (18.6%)	13 (36.1%)	4 (80.0%)	5 (33.3%)	4 (25.0%)
Partial response, *n* (%)	14 (23.7%)	9 (15.2%)	8 (22.2%)	3 (60.0%)	2 (13.3%)	3 (18.8%)
Very good partial response, *n* (%)	7 (11.9%)	2 (3.4%)	3 (8.3%)	1 (20.0%)	1 (6.7%)	1 (6.2%)
Complete response, *n* (%)	2 (3.4%)	0 (0%)	2 (5.6%)	0	2 (13.3%)	0
Minimal response, *n* (%)	16 (27.1%)	12 (20.3%)	10 (27.8%)	1 (20.0%)	4 (26.7%)	5 (31.2%)
Stable disease, *n* (%)	13 (22.0%)	23 (39.0%)	12 (33.3%)	0	6 (40.0%)	6 (37.5%)
Progressive disease, *n* (%)	5 (8.5%)	11 (18.6%)	0	0	0	0
Early death^a^, *n* (%)	2 (3.4%)	2 (3.4%)	1 (2.8%)	0	0	1 (6.2%)

*PR* partial response, *POM* pomalidomide, *CY* cyclophosphamide, *DEX*dexamethasone ^a^Two patients were not available for response due to early death and counted as non-responder

Of 59 patients evaluable during cycle 1–3 at least for one response, 50 were assigned for the addition of cyclophosphamide according to protocol. In total, 36 (61.0%) patients actually received cyclophosphamide. Excluding the two early deaths, *n* = 24 patients showed PD during the first three cycles of which 16 patients received cyclophosphamide and *n* = 24 patients showed SD or MR of which 20 patients received cyclophosphamide. The main reason not to start cyclophosphamide was investigator’s decision in both groups (*n* = 6 and *n* = 3, respectively). This was mainly due to rapid progression together with severe deterioration of the patient, in some patients addition was missed. At start of cyclophosphamide, 16 patients (44.4%) showed PD, 15 patients (41.7%) SD, and 5 patients (13.9%) MR. After addition of cyclophosphamide, 13 patients (36.1%) achieved ≥PR (8 PR, 3 VGPR, and 2 CR). Ten patients (27.8%) showed MR. Of the 16 patients starting cyclophosphamide at primary progression under pomalidomide and dexamethasone, all patients achieved at least SD (5 MR, 3 PR, and 1 VGPR). Of 20 patients with SD or MR after 3 cycles, 9/20 (45.0%) responded with 5 patients achieving a PR, 2 VGPR, and 2 CR. Only patients under the triplet combination achieved a CR.

For those patients (*n* = 13) receiving pomalidomide+dexamethasone without addition of cyclophosphamide, response was documented as follows: 5 PR, 4 VGPR, and 4 MR. Median PFS of the ITT population was 6.4 months, median TTNT 11.0 months, median OS 18.3 months (Fig. 1a–c). Median second PFS from start cyclophosphamide was 4.8 months. Main toxicity was hematologic with neutropenia ≥grade 3 in 66.6%, leukopenia ≥grade 3 in 40.0%, anemia ≥grade 3 in 26.7%, and thrombocytopenia ≥grade 3 in 25.0%. The most commonly reported ≥grade 3 nonhematologic AE was pneumonia in 16.7%.

**Fig. 1:**
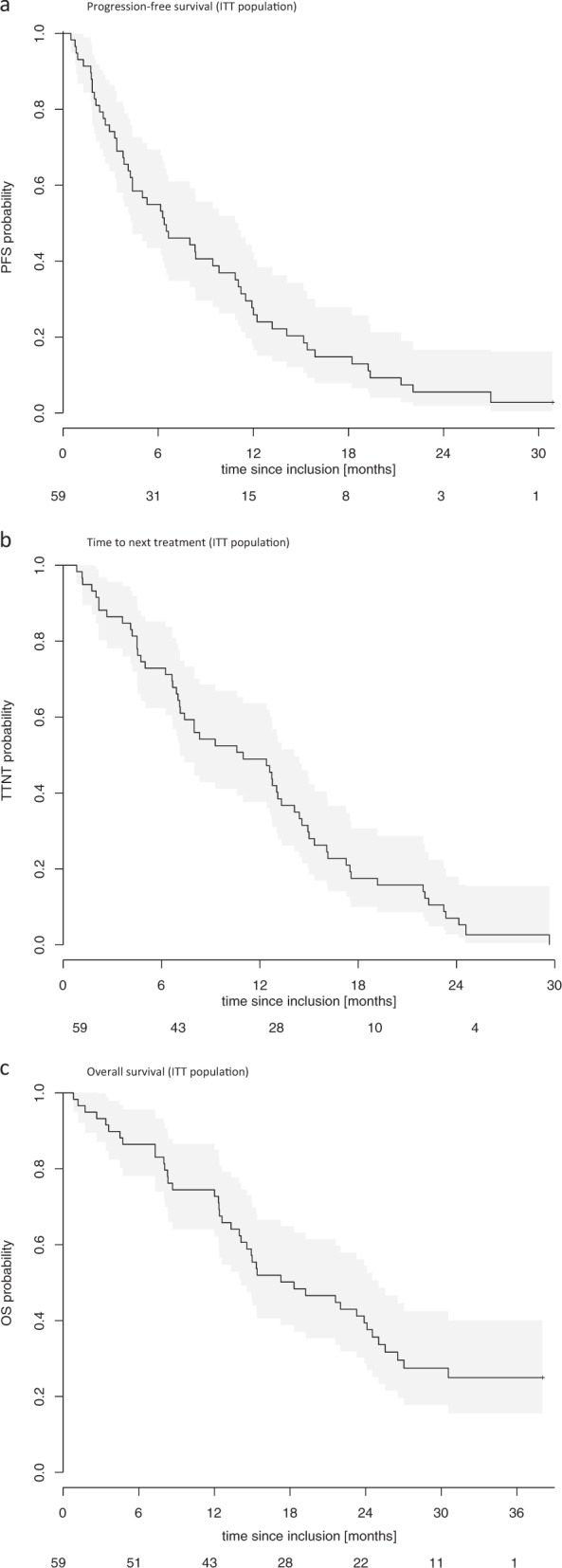
Kaplan Maier estimates for progression-free survival (PFS), time to next treatment (TTNT) and overallsurvival (OS) of the intent to treat (ITT) population. **a** Progression-free survival (ITT population). **b** Time to next treatment (ITT population). **c** Overall survival (ITT population). ITT, intent to treat

In the phase II PERSPECTIVE trial we demonstrated that addition of cyclophosphamide in patients not achieving a PR after three treatment cycles or with primary progression under pomalidomide and dexamethasone was able to rescue a substantial proportion of patients. A conversion into ≥PR was achieved in 36.1% including deep remissions with 5/36 patients achieving a VGPR or CR. Median PFS is 6.4 months and compares favorably with the median PFS reported with pomalidomide and dexamethasone. Of note, some patients initially not responding to pomalidomide + dexamethasone were able to achieve durable responses on the triplet combination with 17/36 patients staying more than 10 additional cycles and 6/36 patients staying more than 20 additional cycles on pomalidomide. While the effect of cyclophosphamide in our trial is clear in patients who experienced a primary progression under pomalidomide + dexamethasone where we could induce in all patients at least an SD, the effect of the third drug is less clear in those patients with a documented SD or MR during the first three cycles as a late response might have been occurred. Moreau et al. showed in the initial pomalidomide + dexamethasone approval trial MM-003 that 17.4% and 13.6% of patients with SD after two and four cycles, respectively, achieved a response during later cycles. With an improvement in reponse of 45.0% (9/20) in patients with SD or MR during the first three cycles including CR and VGPR in the here reported trial, our results indicate a potential benefit of adding cyclophosphamide even in case of early suboptimal response. Furthermore, our trial included a high rate of patients with cytogenetic high-risk disease. It was previously shown that the addition of cyclophosphamide to lenalidomide and dexamethasone might overcome lenalidomide resistance. Here, we demonstrate that the addition of cyclophosphamide is able to overcome resistance to a third-generation immunomodulatory agent. Cyclophosphamide exerts various immunomodulating effects. One potentially important mechanism is the suppression of regulatory T cells^10^. The addition of cyclophosphamide to pomalidomide and dexamethasone was shown to be effective in other trials. Baz et al. reported a randomized phase II trial including 70 patients where pomalidomide + dexamethasone was compared to pomalidomide, cyclophosphamide, and dexamethasone showing a significant increase in ORR, median PFS, and median OS. Our trial was hampered by missed addition of cyclophosphamide in 14 assigned patients either due to protocol violation or due to early and aggressive progression with unability to keep the patient in the protocol. Overall, the triplet combination was feasible with the expected toxicity of the applicated drugs. We saw a potential increase in cytopenias and infections when cyclophosphamide was added to pomalidomide and dexamethasone. Whether the rate of infections was exclusively due to the addition of cyclophosphamide or due to the fact that the inferior, not rapidly responding population was exposed with the triplet regimen and so was kept potentially longer under treatment, cannot be fully differentiated.

Overall, Pomalidomide-based treatment gains in importance due to the emerging use of lenalidomide in frontline treatment. In the current ESMO recommendations, primary extension of pomalidomide and dexamethasone to a triplet is recommended; however, in most countries outside US there is no approved triplet regimen^11^. The triple combination of pomalidomide, cyclophosphamide, and dexamethasone is a cost effective and easy to administer combination treatment for patients with RRMM. In light of the high tolerability and the here observed data in context with the published data and recommendations, we would propose to consider the primary use of the triplet combination rather than to use pomalidomide + dexamethasone alone.

## Supplementary information

Supplemental material - Synopsis of the trial

Supplemental material - Patients characteristics
