# Supplementary material for: Addition of cyclophosphamide on insufficient response to pomalidomide and dexamethasone: results of the phase II PERSPECTIVE Multiple Myeloma trial
Source: Blood Cancer J. 2019 Apr 8;9(4):45. doi: 10.1038/s41408-019-0206-8 (PMC6453945; doi:10.1038/s41408-019-0206-8)
Supplement: Supplementary file 1 — Supplemental material - Synopsis of the trial [file 41408_2019_206_MOESM1_ESM.doc]

# Protocol Outline

| **Title** |
| --- |
| Phase II multicenter, open-label, single arm clinical Study of **P**omalidomide and Dexamethasone  in **R**elap**S**ed myeloma **P**lus r**E**sponse adapted  **C**yclophosphamide as a **T**ailored **I**nno**V**ativ**E** strategy |
| **Short Title** |
| GMMG-PERSPECTIVE |
| **Phase** |
| II |
| **Sponsor** |
| University Hospital Tuebingen,  Geissweg 3  72076 Tuebingen  Germany |
| **Coordinating Investigator (LKP)** |
| PD Dr. med Katja Weisel  University of Tuebingen  Department of Hematology, Oncology, Immunology, Rheumatology und Pulmonology  Otfried-Mueller Strasse 10  72076 Tuebingen  Germany |
| **Financing/ Status of the Sponsor** |
| Non-commercial/Co-financing by pharmaceutical industry |
| **Indication** |
| Relapsed or Refractory Multiple Myeloma |
| **Trial Population** |
| **Inclusion Criteria**  Subjects must satisfy the following criteria to be enrolled in the study:   1. Must be ≥ 18 years at the time of signing the informed consent. 2. Understand and voluntarily sign an informed consent prior to any study related assessments/procedures are conducted. 3. Able to adhere to the study visit schedule and other protocol requirements. 4. Subjects must have documented diagnosis of multiple myeloma and have measurable disease (serum M-protein ≥ 0.5 g/dL or urine M-protein ≥ 200 mg/24 hours). In case of oligosecretory myeloma: involved FLC level ≥ 10 mg/dl, provided sFLC ratio is abnormal. 5. Subjects must have had at least two prior anti-myeloma regimens (incl. bortezomib and lenalidomide) and must have been progressed under the last prior treatment. Induction therapy followed by ASCT and consolidation/ maintenance will be considered as one regimen. 6. ECOG performance status score of 0, 1, or 2. 7. Females of childbearing potential (FCBP^1^) must agree:  - to utilize two reliable forms of contraception simultaneously or practice complete abstinence from heterosexual contact for at least 28 days before starting study drug, while participating in the study (including dose interruptions), and for at least 28 days after study treatment discontinuation and must agree to regular pregnancy testing during this timeframe - to abstain from breastfeeding during study participation and 28 days after study drug discontinuation.  1. Males must agree:  - to use a condom during any sexual contact or practice complete abstinence from heterosexual contact with a pregnant female and a FCBP while participating in the study, during dose interruptions and for 28 days following discontinuation from this study, even if he has undergone a successful vasectomy - to refrain from donating semen or sperm while on Pomalidomide and for 28 days after discontinuation from this study treatment.  1. All subjects must agree to refrain from donating blood while on study drug and for 28 days after discontinuation from this study treatment. 2. All subjects must agree not to share medication.   ^1^A female of childbearing potential is a sexually mature woman who: 1) has not undergone a hysterectomy or bilateral oophorectomy; or 2) has not been naturally postmenopausal for at least 24 consecutive months (i.e., who has had menses at any time in the preceding 24 consecutive months; amenorrhea following cancer therapy does not rule out childbearing potential). |
| **Exclusion Criteria**  The presence of any of the following will exclude a subject from enrolment:   1. Any of the following laboratory abnormalities:  - Absolute neutrophil count (ANC) < 1,000/μL. - Subject with platelet count ≤ 30,000/µL are not eligible regardless of the percentage of plasma cells in the bone marrow. For subject with platelet count > 30,000/µL and < 75,000/µL, percentage of plasma cells in bone marrow should be ≥ 50%. - Corrected serum calcium > 14 mg/dL (> 3.5 mmol/L). - Hemoglobin < 8 g/dL (< 4.9 mmol/L; prior RBC transfusion or recombinant human erythropoietin use is permitted). - Serum SGOT/AST or SGPT/ALT > 3.0 x upper limit of normal (ULN) except due to multiple myeloma. - Serum total bilirubin > 2.0 mg/dL (34.2 μmol/L); or > 3.0 x ULN for subjects with hereditary benign hyperbilirubinemia. - GFR < 30 ml/min or patient requiring hemodialysis  1. Prior history of malignancies, other than MM, unless the subject has been free of the disease for ≥ 5 years. Exceptions include the following:  - Basal or squamous cell carcinoma of the skin - Carcinoma in situ of the cervix or breast - Incidental histological finding of prostate cancer (TNM stage of T1a or T1b).  1. Previous therapy with Pomalidomide. 2. Hypersensitivity to thalidomide, lenalidomide, or Dexamethasone (this includes ≥ Grade 3 rash during prior thalidomide or lenalidomide therapy). 3. Peripheral neuropathy ≥ Grade 2. 4. Subjects who received an allogeneic bone marrow or allogeneic peripheral blood stem cell transplant less than 12 months prior to initiation of study treatment and who have not discontinued immunosuppressive treatment for at least 4 weeks prior to initiation of study treatment and are currently dependent on such treatment. 5. Subjects who are planning for or who are eligible for stem cell transplant. 6. Subjects with any one of the following:  - Congestive heart failure (NY Heart Association Class III or IV) - Myocardial infarction within 12 months prior to starting study treatment - Unstable or poorly controlled angina pectoris, including Prinzmetal variant angina pectoris.  1. Subjects who received any of the following within the last 14 days of initiation of study treatment:  - Major surgery (kyphoplasty is not considered major surgery) - Use of any anti-myeloma drug therapy.  1. Use of any investigational agents within 28 days or five half-lives (whichever is longer) of treatment. 2. Incidence of gastrointestinal disease that may significantly alter the absorption of Pomalidomide. 3. Subjects unable or unwilling to undergo antithrombotic prophylactic treatment. 4. Any serious medical condition, laboratory abnormality, or psychiatric illness that would prevent the subjects from signing the informed consent form. 5. Pregnant or breastfeeding females. 6. Known human immunodeficiency virus (HIV) positivity, active infectious hepatitis A, B or C or chronic hepatitis B or C. 7. Any condition that confounds the ability to interpret data from the study. |

| **Objectives** |
| --- |
| **Primary Objective/ Endpoint**   - To determine response rate |
| **Secondary Objectives/ Endpoints**   - To determine progression free survival of Pomalidomide in combination with low-dose Dexamethasone and intravenous Cyclophosphamide in case of suboptimal response or first evidence of progressive disease in subjects with relapsed or refractory MM - To evaluate safety: incidence of Second Primary Malignancies (SPM) (according to CTCAE 4.0: hematological toxicity, sensoric neuropathy, infections > CTC III) - To evaluate overall survival - To assess time to subsequent therapy - To assess time to myeloma response - To assess cytogenetic profile |
| **Trial Design** |
| Phase II multicenter, open-label, single arm clinical study. |
| **Investigational Medicinal Products:** |
| 1) Pomalidomide administered orally at the starting dose of 4 mg/day on Days 1-21 of a 28-day cycle  2) Low-dose Dexamethasone administered orally at the starting dose of 40 mg/day (≤ 75 years old) or 20 mg/day (> 75 years old) on Days 1, 8, 15, and 22 of a 28-day cycle  3) Cyclophosphamide administered intravenously 500 mg/m² on Days 1 and 15 of a 28-day cycle |
| **Sample Size**  60 patients |
| **Statistical Analysis** |
| The GMMG-PERSPECTIVE is designed to determine the objective response rate (ORR), defined as the portion of patients with partial responses (PRs) or better as best response during treatment period (maximum two years) in the combined concept of Pomalidomide/Dexamethasone and Cyclophosphamide after.  In the included patient population, an ORR of ~30% is estimated after 3 cycles and as the best ORR of Pomalidomide/Dexamethasone. Calculating 10% of primary refractory patients and/or drop-outs due to toxicities, ~60% of patients will receive Cyclophosphamide after 3 cycles due to suboptimal response or during cycle 2 and 3 in case of first evidence of disease progression . It is estimated, that 30% of these patients (18% of total patient population) will achieve at least PR as best response during treatment.  Hypothesis regarding secondary endpoint:  Addition of Cyclophosphamide in the above setting will lead to a PFS of 6.0 months for the overall study population. |
| **Trial Duration and Dates** |
| Total trial duration: *[36 months]*  Duration of the clinical Phase: *[24 months]*  Beginning of the preparation Phase: *[Q1 2013]*  FSI (First Subject In): *[Q2 2014]*  LSI (Last Subject In): *[Q2 2015]*  LSO (Last Subject Out): *[Q4 2015]*  DBL (Data Base Lock): *[Q3 2016]*  Statistical Analyses Completed: *[Q4 2016]*  Trial Report Completed: *[Q2 2017]* |
