# Supplementary material for: Addition of cyclophosphamide on insufficient response to pomalidomide and dexamethasone: results of the phase II PERSPECTIVE Multiple Myeloma trial
Source: Blood Cancer J. 2019 Apr 8;9(4):45. doi: 10.1038/s41408-019-0206-8 (PMC6453945; doi:10.1038/s41408-019-0206-8)
Supplement: Supplementary file 2 — Supplemental material - Patients characteristics [file 41408_2019_206_MOESM2_ESM.docx]

Table 1. Patient characteristics

| Characteristic | Overall  (n = 59) |
| --- | --- |
| Age, median (range), years | 67 (47-81) |
| Male, n (%) | 27 (45.8%) |
| Number of prior lines, median (range) | 3 (2-5) |
| Prior lenalidomide, n (%) | 59 (100%) |
| Prior bortezomib, n (%) | 59 (100%) |
| Prior cyclophosphamide, n (%) | 50 (84.8%) |
| Prior melphalan, n (%) | 56 (94.9%) |
| Prior thalidomide, n (%) | 12 (20.3%) |
| Prior autologous SCT, n (%) | 47 (79.7%) |
| Prior allogeneic SCT, n (%) | 4 (6.8%) |
| ECOG PS 0/1/2/ unknown, n | 23/27/6/3 |
| ISS stage I/II/III/ unknown, n | 18/17/19/5 |
| Cytogenetic profile* |  |
| High-risk (Del 17p**, t(4;14), > 3 copies 1q21, n (%) | 17 (43.6%) |
| Standard risk, n (%) | 22 (56.4%) |
| missing, n | 20 |

*Evaluable in 39 patients

**Positive when in > 10% of nucleated cells

SCT = stem cell transplantation, PS = performance status
